# Is vigilance a personality trait? Plasticity is key alongside some contextual consistency

**DOI:** 10.1371/journal.pone.0279066

**Published:** 2022-12-12

**Authors:** Claudia Mettke-Hofmann

**Affiliations:** School of Biological and Environmental Sciences, Liverpool John Moores University, Liverpool, United Kingdom; University of Pretoria, SOUTH AFRICA

## Abstract

Animals regularly scan their environment for predators and to monitor conspecifics. However, individuals in a group seem to differ in their vigilance linked to age, sex or state with recent links made to personality. The aims of the study were to investigate whether a) individuals differ consistently in their vigilance, b) vigilance is linked to other personality traits and c) other factors affect vigilance in the colour polymorphic Gouldian finch. Birds were tested in same (red-headed or black-headed) or mixed head colour morph same sex pairs in four contexts (novel environment, familiar environment, two changed environments). Vigilance was measured as horizontal head movements. Vigilance showed contextual consistency but no long-term temporal consistency over a year. Head movements were only weakly linked to other personality traits indicative of a risk-reward trade-off with more explorative individuals being less vigilant. Vigilance was highly plastic across situations and affected by group composition. Mixed head colour morph pairs made more head movements, potentially linked to higher social vigilance. Results indicate that vigilance is a highly plastic trait affected by personality rather than a personality trait on its own, which allows adapting vigilance to different situations.

## Introduction

Animals routinely interrupt their current behaviour to scan their environment for potential threats, particularly from predators. Grouping helps to reduce the costs of vigilance to detect predators [1–4] but increases demand on monitoring conspecifics (social vigilance; [3, 5–8]). However, there is increasing evidence that individuals in a group differ in their vigilance due to differences in state, sex or age (e.g., [2, 9, 10]).

Vigilance is often measured in a foraging context to investigate trade-offs between foraging and vigilance [11] by assessing the frequency and duration of heads-up (e.g., [4, 9, 11, 12]). More recently, primarily horizontal head movements (in contrast to primarily vertical head movements) have been used to measure vigilance. This method allows measuring vigilance in any context and provides more information about the strategies used when scanning the environment [13]. Every horizontal movement of the head brings the eyes of an individual in contact with a different part of the environment [13], hence the frequency of head movements indicates how much of its environment an animal covers within a given time [13]. A high frequency of head movements means that different areas of the environment are scanned in fast succession with a short duration of looking in a particular direction. This has been linked to a visual search strategy that allows detecting any threats quickly [14] and often occurs in more dangerous situations (e.g., novel environments, at the periphery of a group, in small groups; [15–17]). In contrast, a low frequency of head movements indicates a longer dwelling on one aspect of the environment consistent with a visual tracking strategy that allows collecting information about distance, identity and movement of a target (e.g., predators or conspecifics; [14]). Both methods (heads-up and horizontal head movements) are consistent in that a higher frequency of vigilance (more heads-up or more horizontal head movements) usually occurs in more dangerous situations and results in a faster detection of predators [13].

Differences in vigilance between individuals in a group can be linked to their vulnerability; females in size dimorphic species are more vigilant than males [10] as are individuals at the periphery of groups [18]. Whereas age seems to follow an inverse U-shape curve with juveniles [9, 19, 20] and old adults [4, 21] being less vigilant, potentially reflecting the need for higher food intake in juveniles, whereas bigger size or senescence may be the reason in adults.

Another factor differently affecting individual’s vigilance is group composition. Vigilance in groups with young is higher than without young [18], particularly in females with young [5, 8, 10, 20]. Moreover, in ibex (*Capra sibirica*) female groups and mixed sex groups were more vigilant than male groups reflecting the higher predation in female groups and social vigilance directed towards harassing males in mixed groups [4]. Higher vigilance has also been observed in colour polymorphic species when in mixed morph groups as compared to monomorphic groups again indicating social vigilance [22].

More recently, vigilance has been linked to consistent individual differences (personality). While there is considerable plasticity in vigilance across situations (higher vigilance in novel situations or under higher predation risk [10, 18, 23, 24]), habitats [25] and season [2–4], individuals differ consistently in their vigilance over time [19, 26–29]. Moreover, a few studies found vigilance to be consistent within [19] and across contexts [24, 29].

The above findings suggest that vigilance is a personality trait on its own. Whereas other studies have treated vigilance more as a (cognitive) trait that may be affected by personality (measured as boldness, aggression etc) of an individual [30, 31]. In brief, personalities may vary along a risk-reward continuum with bold, aggressive individuals prioritising fast and high rewards over risk, whereas shy, non-aggressive individuals prioritise safety over rewards [32]. Consequently, bold individuals would be expected to be less vigilant. This has been confirmed in a range of species from birds to mammals including humans [26, 28, 30, 33], whereas in three-spined sticklebacks (*Gasterosteus aculeatus*) vigilance differed consistently between individuals but was unrelated to boldness [31]. In Eurasian siskins (*Carduelis spinus*) bolder individuals had a higher scan rate indicating higher vigilance compensating for their riskier behaviour [34]. In the human literature another personality trait–extraversion/ introversion has been weakly but consistently linked to vigilance with extroverts being less vigilant [33, 35, 36]. Finally, Risko et al. [37] found that in humans, perceptual curiosity (the desire to touch something novel as part of the openness dimension) was positively linked to vigilance.

Here we investigate vigilance in the colour-polymorphic Gouldian finch (*Chloebia gouldiae*). The species occurs in two main head colours, black and red, in the wild with about 70% black-headed birds and 30% red-headed birds and less than 1% yellow-headed birds in most populations [38]. They are highly social and inhabit tropical savannah grassland in North Australia [39]. Their personality is linked to their head colour with red-headed birds being more aggressive but less explorative towards a novel object and risk-averse in potentially dangerous situations than black-headed birds [40]. While the two main morphs do not differ in their vigilance, group composition matters. Whenever red-headed birds are present (mixed or pure red-headed groups), vigilance is heightened compared to pure black-headed groups [22]. Moreover, vigilance was highly plastic across situations with fewer head movements the more novel a situation was [22].

The aim of the current study was to investigate

1. Whether vigilance is a personality trait (contextual and temporal consistency).
2. Whether vigilance correlates with other personality traits.
3. How vigilance changes in relation to situation, age, sex, head colour and head colour composition.

## Material and methods

Twenty-two Gouldian finches took part in the experiment with 12 females (6 black-headed, 6 red-headed) and 10 males (4 black-headed, 6 red-headed). Ages ranged from 1 to 8 years. The birds originated from 10 different bird breeders acquired over several years with all birds residing in the Animal Facility at Liverpool John Moors University since at least a year.

Birds were kept in mixed age, sex and head colour groups of about 6 birds, each. Dimensions of the holding cages were 120 x 80 x 100 cm (length x depth x height) and consisted of three closed walls with a wire mesh front and ceiling. The interior consisted of natural twigs and perches with food provided in feeders at the front. Food consisted of a mixture of Blattner Amadine Zucht Spezial (Gouldamadine), Blattner Astrilden Spezial and Blattner rote Mannahirse (Blattner Heimtierfutter, Ermengerst, Germany). Blattner bird grit was provided separately, as were eggshells. Cages contained water dispensers and a bath. The light regime was 13 h light to 11 h dark.

### Experimental procedure

Experiments were conducted in an adjacent room containing six cages (120 x 70 x 100 cm) arranged back-to-back in two rows. Cages consisted of three wooden walls with a wire mesh front and ceiling facilitating that birds could not see but hear each other. Two perches were available left and right running perpendicular to the front. Food and water were provided at the front wire. A camera was permanently mounted on a tripod one metre away from the front of each cage.

For the current experiment only four of the six cages were used due to logistical reasons. Experiments were conducted over a seven-day period testing four groups at the same time (= one batch) with the next batch of birds being moved into the experimental cages the day after the preceding group had finished. Cages were thoroughly cleaned between groups. Gouldian finches are highly social; hence, birds were tested in same sex pairs either in same or mixed head colour pairs (4 black-headed, 4 red-headed, 4 mixed head colour pairs balanced across sexes). As there were only 22 birds, two black-headed males were used a second time to provide a partner for two red-headed males. Vigilance data of the re-used birds were not included in the analysis as they were already represented in the data set with their first testing. Head colour combinations and sexes were balanced within and across batches.

The following experiments were conducted: Vigilance was investigated in four different situations. (1) novel environment (when released into the experimental cage), (2) familiar environment (day 5 in experimental cage), (3) changed environment 1 (day 6) and (4) changed environment 2 (day 7). A change in the environment consisted of either a novel object (metal star, 10.5 x 4.5 cm, height x width) placed at a neutral location in the cage (over a perch but away from food and water) or a novel object (Christmas stocking sock, 11.5 x 5.5 x 2.5 cm; HxWxDepth) placed over the feeder. The order of presentation (day 6 or 7) was balanced across the objects. The two changed environments represented different levels of risk. The star at the neutral location could be ignored when wanted, causing a lower level of risk, whereas the sock over the feeder required interaction and approach when the birds wanted to feed. All experiments started at 10:00am and lasted for one hour. Data were recorded with digital video cameras using the GeoVision 1480 software (GeoVision Inc., Taipei, Taiwan) for later analysis.

Sixteen of the 22 birds had taken part in a very similar vigilance experiment a year earlier with exactly the same procedure for the novel and familiar situation but a slightly different protocol for the changed situation. Instead of placing a novel object inside the cage, a novel object (toy penguin or panda bear, 15–17 cm height, respectively) mounted on a tripod was placed 40 cm away from the front wire outside the cage [22]. Experiences with the cages a year ago were unlikely to have affected responses as reactions to unfamiliar situations usually fully recover after non-exposure of three to four weeks [41, 42].

Birds were released into the cage with video recording starting immediately to collect vigilance data in the novel environment (situation 1). After one hour, video recording was stopped, and the birds had until day 5 to habituate to the cage. On day 5, vigilance was recorded again in the now familiar environment (situation 2). The next day, either the star was positioned over the neutral perch or the stocking sock over the feeder (changed environment one and two–situation 3 and 4). In case of the stocking sock, birds were food deprived for one hour prior to the start of the experiment to have similar hunger levels. On day 7, the object not used on day 6 was introduced. On both days recording lasted for one hour after which the objects were removed. Birds were moved back into their holding cages after the experiment had finished on day 7.

### Data analysis

Vigilance was measured as horizontal head movements [13] and defined as any visible movement of the head. Birds rarely looked up or down and these head movements were not considered. Frequency of head movements was extracted for each individual and situation. This measure is inversely related to the duration of looking into a particular direction [17].

All analyses were conducted with SPSS v. 26. The full data set is available in the S1 Table. To answer the first question whether vigilance is a personality trait, I first tested for contextual consistency of vigilance across the four situations in all birds (n = 22) using Pearson correlation tests. In a second step, I tested for contextual and temporal consistency across a year and three situations (based on the outcome of the correlation analysis) using the birds that were tested twice one year apart (n = 16). Vigilance in the novel, familiar and changed situation in the two years was compared. The changed situation presented a change that could be ignored (away from food and water) and differed slightly between years. In year two it was the star at a neutral location, whereas in year one an object was positioned outside the cage. Two objects were used in year one and the mean of the vigilance shown across the two objects was used in the analysis [22]. This analysis was done with One-Way ANOVA. One-Way ANOVAs compare within group variation against between group variation. Here intra-individual variation in vigilance across different situations and years was compared against the between-individual variation in vigilance. Each individual contributed six data points (96 data points in total).

The second question was about whether vigilance correlates with other personality traits. Data from the birds in year two (n = 22) in the two changed situations were used for this analysis. The star positioned at a neutral location is a standard measure for object exploration [43]. The novel object elicits both, approach (neophilia) and avoidance (neophobia) reactions. Approach occurs when the motivation to approach is higher than the motivation to avoid the novel object [43]. Object exploration has been shown to be consistent over time and a personality trait in the Gouldian finch [40]. Three latencies were recorded: the time between introducing the object over the perch and a) landing on the perch with the novel object, b) being in reach (two body lengths marked with a black line on the perch) of the object and c) touching the object. Only the latency to land on the perch was used for further analysis as all but two birds landed on the perch (20/22) providing the most variation, whereas 12 approached the object in reach and nine touched the object. Latency to perch was positively related with latency in reach (Spearman correlation: n = 22, corr. coef. = 0.58, p = 0.005) and latency in reach with latency to touch (corr. coef. = 0.75, p < 0.001).

The stocking sock positioned above the feeder is a standard measure for object neophobia [43, 44]. Here the motivation to feed and the motivation to avoid the novel object are in conflict with each other and approach times are a good representation of the fear involved [43]. Object neophobia is a consistent trait and positively correlated with object exploration in the Gouldian finches [45]. Again, three latencies were recorded: the time between positioning the novel object on top of the feeder and a) being in reach of the feeder, b) landing on the feeder and c) feeding. Only the latency to be in reach of the feeder was used for further analysis as ten out of twenty-two birds approached the feeder, whereas nine landed on the feeder and one fed. The latency to be in reach of the feeder was highly correlated with the latency to land on the feeder (Spearman Correlation: n = 22, corr. coef. = 0.96, p < 0.001) and close to significance with the latency to feed (corr. coef. = 0.39, p = 0.069). The latency to land on the perch with the object (object exploration) and the latency to be in reach of the feeder (object neophobia), respectively, were correlated with the vigilance measure for each situation using Spearman correlations as data deviated from normality.

To address the third question about how situation, age, sex, head colour and head colour composition affect vigilance, I ran General Linear Mixed Models (GLMM). Analyses were run on the individual level with situation as repeated measure and individual as random factor to account for repeated testing. The sample size was n = 22 with each bird contributing four data points, one for each situation, resulting in 88 data points in total. The dependent variable was frequencies of head movements with an identity link function. A series of models were built to test for effects of different variables and interactions. The basic model consisted of four fixed factors (situation, head colour morph, sex and age class), one covariate (partner head colour morph) and two interaction terms (head colour morph x partner head colour morph and head colour morph x sex), which is the same basic model used for analysis in the vigilance study the year before [22] except for sex being a fixed factor now rather than a covariate. Using the same basic model allows direct comparison with the new situations used in the current study. Age classes consisted of one- to two-year-old birds (n = 5), three to four years of age (n = 8) and older than four years (n = 9). More complex models included interaction terms with situations and combinations of two interactions. Akaike criterion was used to select the best model.

### Ethical note

Experiments were conducted in accordance with The Association for the Study of Animal Behaviour (ASAB) ethical guidelines [46] and were non-invasive. Experiments were approved by the University Ethics Committee.

## Results

Question 1 investigated consistency of vigilance across the four situations. Head movements in the familiar environment were positively correlated with head movements in one of the changed (star at a neutral location) and the novel environment situation (Pearson correlation: n = 22, familiar vs changed (star at neutral location) corr. coef. = 0.68, p < 0.001, familiar vs novel corr. coef. = 0.50, p = 0.017). Head movements in both changed environments showed a weak trend to be positively correlated with head movements in the novel environment (star: corr. coef. = 0.37, 0.092, sock: corr. coef. = 0.37, p = 0.089).

Furthermore, temporal and contextual consistency was investigated in a subset of birds that were tested in this study and the year before considering vigilance in the familiar situation, the novel situation and one changed situation (novel object at a neutral location or outside the cage in year one). Within individual variation did not differ significantly from the between individual variation (One-Way ANOVA: n = 16, F = 1.365, p = 0.185).

Question 2 was concerned about a link between vigilance and other personality traits. Head movements in the changed situation (sock above feeder) correlated positively with the latency to land on the perch with the novel object (corr. coef. = 0.53, p = 0.011) but no other correlations were identified (all p > 0.2).

**Fig 1:**
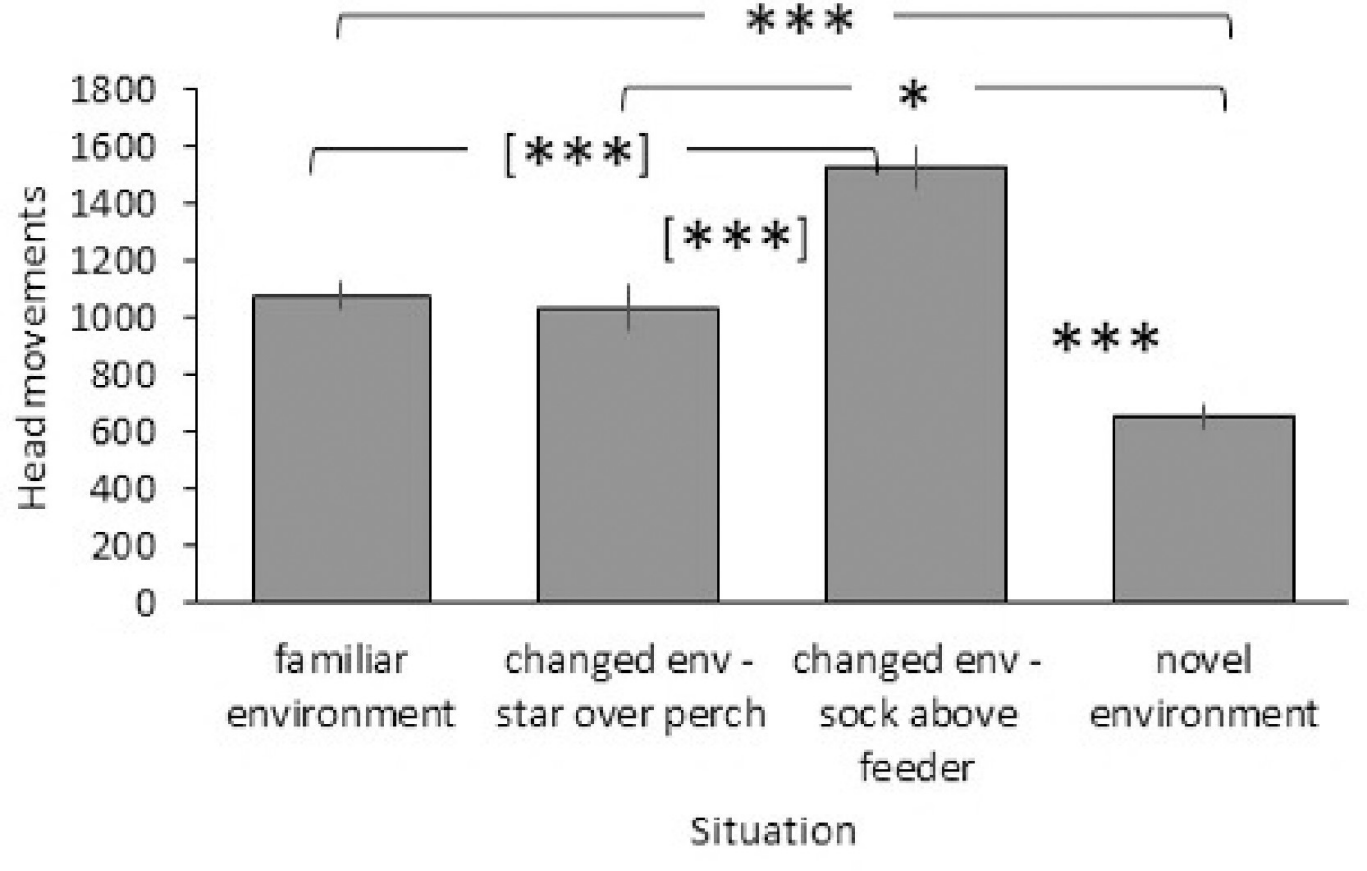
Plasticity in vigilance across situations. Mean and standard error of frequency of head movements per hour (n = 22) in four situations: familiar environment, two changed environments (star over neutral perch, sock above feeder) and novel environment. * P < 0.05; *** P < 0.001; [***] P < 0.001 from additional paired t-tests.

Question 3 addressed how vigilance in the different situations was affected by head colour morph, age, sex and head colour morph composition. The best model retaining the most information was the basic model with the interactions age class x situation, sex x situation, head colour morph x situation and partner head colour morph x situation (Table 1). The following variables and interactions were significant (Table 2). Vigilance differed across situations (F_3,62_ = 44.421, p < 0.001; Fig 1) with the fewest head movements in the novel environment (novel vs familiar t_21_ = 3.712, p < 0.001, novel vs changed (neutral location) t_21_ = 2.038, p = 0.046, novel vs changed (above feeder) t_21_ = 5.010, p < 0.001). Head movements were intermediate in the familiar environment and were significantly higher than in the novel environment (see above), but significantly lower than in the changed environment with the object above the feeder (statistics are from an additional paired t-test as the GLMM only provided outputs in relation to the novel situation: t_21_ = -4.750, p < 0.001). Head movements in the familiar and changed environment with the object at a neutral location were similar (additional paired t-test: t_21_ = 0.926, p = 0.365). Finally, head movements were highest in the changed environment with the object above the feeder and differed significantly from all other situations (additional paired t-test: changed (object above feeder) vs changed (object at neutral location) t_21_ = -5.062, p < 0.001, for all other comparisons see above).

**Table 1:** Model selection outcome using Akaike information criterion (AIC) comparing vigilance in Gouldian finches with respect to head colour, partner head colour, age class and sex in different situations.

Model	AIC	Diff^1^
Basic model^2^ + age class x situation + sex x situation + head colour x situation + partner head colour x situation	917.785	
Basic model + age class x situation + sex x situation + head colour x situation	954.220	36.435
Basic model + age class x situation + sex x situation + partner head colour x situation	956.172	38.387
Basic model + sex x situation + head colour x situation + partner head colour x situation	989.993	72.208
Basic model + age class x situation + head colour x situation	991.324	73.539
Basic model + age class x situation + sex x situation	991.369	73.584
Basic model + age class x situation + partner head colour x situation	993.544	75.759
Basic model + sex x situation + head colour x situation	1026.175	108.39
Basic model + age class x situation	1028.228	110.443
Basic model + sex x situation + partner head colour x situation	1029.487	111.702
Basic model + head colour x situation	1063.747	145.962
Basic model + sex x situation	1064.390	146.605
Basic model + partner head colour x situation	1067.053	159.268
Basic model	1101.648	183.863

^1^Diff: Difference in AIC to best model (italics)

^2^Basic model: Situation + age class + sex + colour morph + partner colour morph (C) + colour morph x partner colour morph + colour morph x sex

C: variable entered as covariate

**Table 2:** General Linear Mixed Model outcome of best model (see Table 1).

Variables	F	Df1	Df2	P
**Corrected model**	9.756	25	62	<0.001
**Situation**	44.421	3	62	<0.001
**Age class**	5.330	2	62	0.007
**Sex**	5.012	1	62	0.029
**Colour morph**	0.035	1	62	0.853
**Colour morph x partner colour morph**	16.164	2	62	<0.001
**Colour morph x sex**	1.835	2	62	0.180
**Age class x situation**	0.326	6	62	0.921
**Sex x situation**	1.648	3	62	0.188
**Colour morph x situation**	1.178	3	62	0.325
**Partner colour morph x situation**	0.303	3	62	0.823

**Fig 2:**
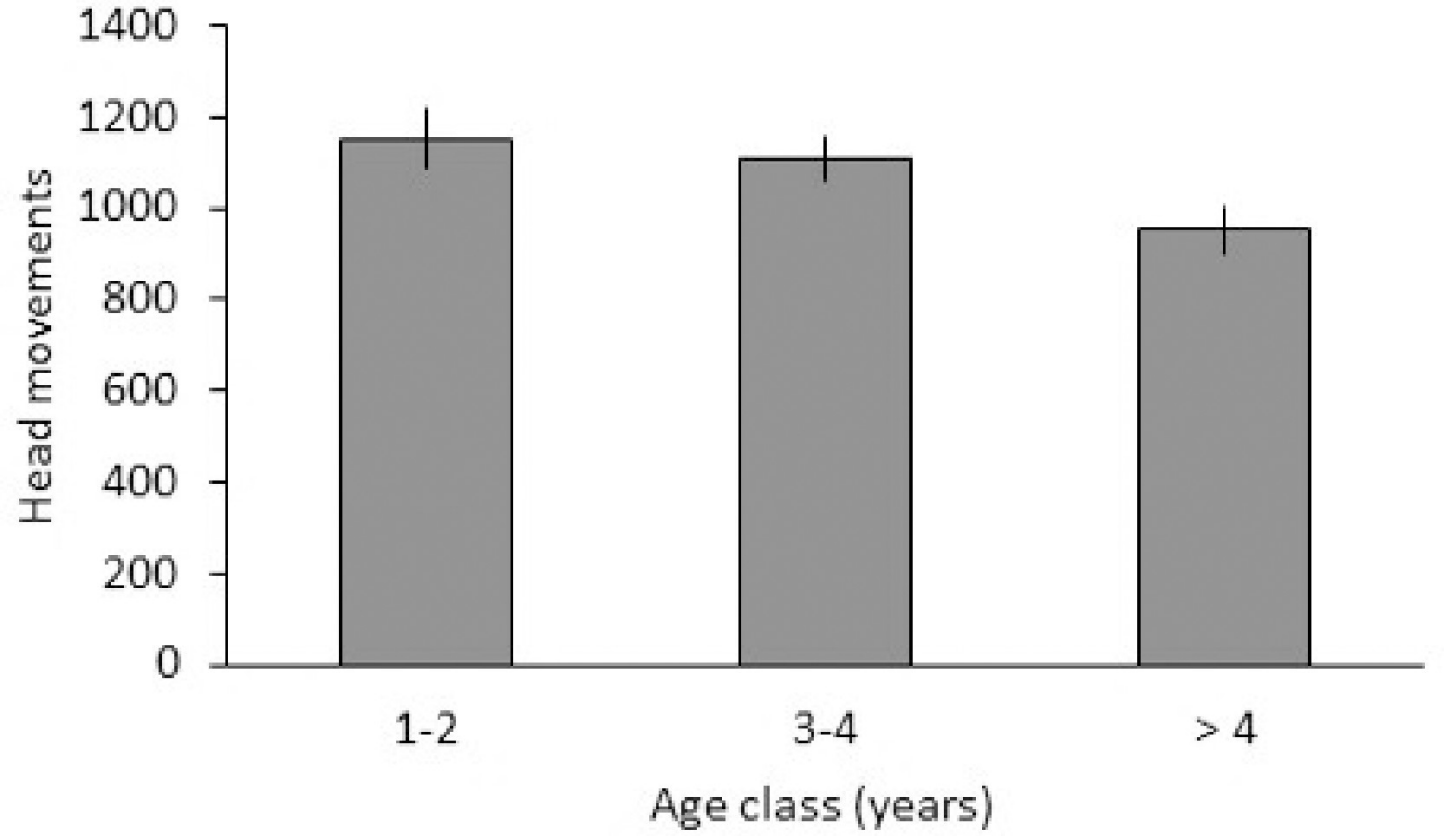
Age effects on vigilance. Mean and standard error of frequency of head movements per hour (n = 22) across age classes.

Age class was the second main factor with a significant effect (F_2,62_ = 5.339, p = 0.007; Fig 2). Overall, head movements decreased with age, particularly in the oldest age class. However, posthoc tests were not significant (1–2 years vs > 4 years: t = 1.141, p = 0.258, 3–4 years vs > 4 years: t = 1.425, p = 0.159).

**Fig 3:**
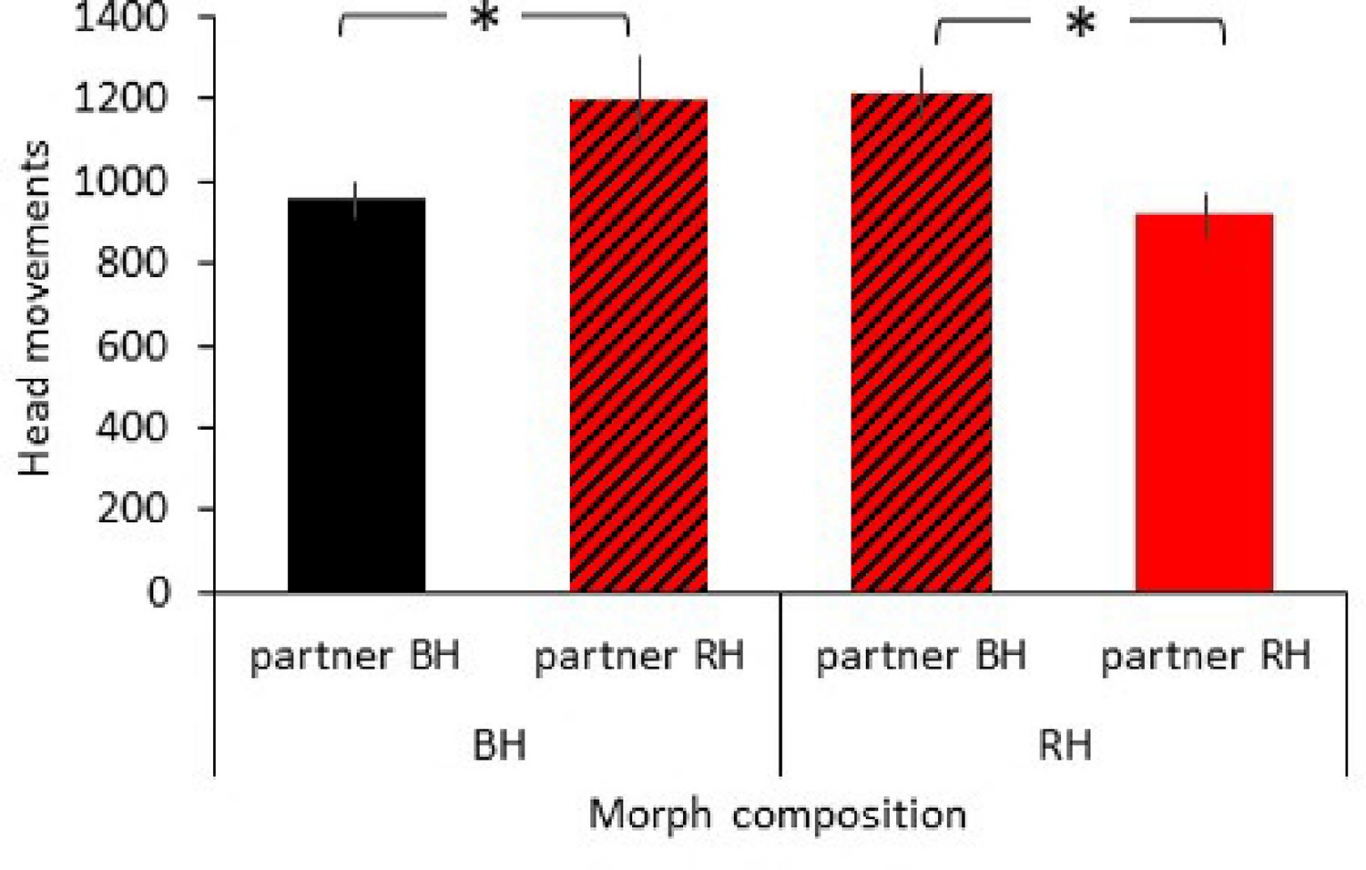
Effect of group composition on frequency of head movements. Mean and standard error of frequency of head movements per hour (n = 22) for black-headed (BH) and red-headed (RH) birds when in pure black-headed (black bar), red headed (red bars) or mixed head colour (hatched) morph pairings. * P < 0.05.

The main factor sex had a significant effect (F_1,62_ = 5.012, p = 0.029) with males making fewer head movements (1012 ± 48) than females (1132 ± 41). Moreover, the interaction head colour morph x partner head colour morph was significant (F_2,62_ = 16.164, p < 0.001; Fig 3). Mixed head colour morphs made significantly more head movements than pure head colour morphs (pure black-headed vs mixed head colour: t = -2.540, p = 0.014, pure red-headed vs mixed head colour: t = 2.277, p = 0.026). No other variables and interactions were significant. The random effect of individual was not significant (z = 0.077, p = 0.939).

## Discussion

Vigilance measured as the frequency of head movements showed some short-term contextual consistency between three situations (familiar–changed (object at neutral location)–novel environment) but no long-term temporal consistency across one year. Moreover, head movements showed some weak links to other personality traits. Vigilance was plastic across situations, decreased with age and was affected by group composition.

Vigilance showed short-term contextual consistency. Specifically, head movements in the familiar environment correlated with head movements in the novel environment and the changed environment with a novel object at a neutral location. This corroborates findings in junglefowl (*Gallus gallus*), which showed contextual consistency in vigilance between a novel environment and a novel object placed at a neutral location in the arena [29]. In hyaenas (*Crocuta crocuta*) vigilance was consistent within contexts (resting, feeding, nursing) but not between contexts [19]. Interestingly, vigilance in the changed situation with the object above the feeder did not correlate with vigilance in any of the other situations. This is surprising and requires further investigation. This was the only situation, in which a change occurred around an important resource. The motivation to feed may have affected vigilance, particularly as birds were food deprived. Indeed, some individuals became very agitated, moved a lot and showed high vigilance up to the end, whereas others gave up on the food and settled down after a while with fewer head movements.

Vigilance showed no long-term consistency (over one year). This contrasts with other studies, which found temporal consistency in hyaenas [19] and orange-winged amazons (*Amazona amazonica*) over one year [28], in redshanks (*Tringa tetanus*) over a 3-month period [26] and in cliff swallows (*Petrochelidon pyrrhonata*) within a breeding season [27]. This indicates that Gouldian finches show short-term contextual consistency but no long-term temporal consistency. One reason for the lack of long-term consistency could be that birds were tested with different partners as group composition has been shown to affect vigilance [e.g., 4, 18, 22]. Future studies should test to which extent group composition affects consistency and whether vigilance is consistent over time.

Vigilance was a poor predictor of other personality traits with only the frequency of head movements in the changed situation with the object above the feeder (neophobia situation) correlating with the latency to land on the perch with the novel object (exploration situation). This may indicate that highly vigilant individuals are also more fearful and consequently hesitate longer to engage in exploration than less vigilant individuals. In orange-winged amazons, neurotic birds (more anxious and vulnerable to stress) were more vigilant than less anxious [28]. It also weakly links to the risk-reward trade-off hypothesis that risk-takers are less vigilant [26, 30, 47]. However, it is unclear why this link was only found across contexts rather than within the same context with the object above the feeder. Generally, vigilance was not directly linked to the latencies to explore and or to approach the food (neophobia). This is in agreement with Zidar et al. [29] who did not find a link between vigilance and the latency to approach a novel object in a slightly novel environment in junglefowl. Likewise, the number of different foraging areas used (i.e., exploration of novel habitats) did not correlate with vigilance in redshanks [26]. Most studies so far found a link between vigilance and the personality trait extraversion (sociability) with more extrovert and social individuals being less vigilant [28, 33, 35, 36]. Sociability has not been considered in this study but should be included in future studies. Taken together, vigilance is a trait than can be affected by personality rather than a personality trait on its own.

Vigilance showed considerable plasticity across situations, which indicates that individuals adapt their vigilance to the degree of threat/ danger perceived. Such plasticity is well documented in a range of taxa in birds [22, 24, 26, 48] and mammals [2, 7, 20, 30, 49, 50]. Birds were most vigilant when a change occurred around an important resource (novel object above feeder). This indicates that they perceived this change as highly threatening. Higher vigilance often occurs in more risky situations e.g., when at the periphery of a flock [51], in a novel situation [23, 52] or once a predator has been detected [24, 53, 54]. Interestingly, a novel object at a neutral location did not increase vigilance as compared to the familiar situation. This is surprising as an earlier study found reduced vigilance when an object was positioned outside the cage [22]. This was interpreted as a more intense assessment of the novel object with spending more time assessing the novel object and consequently fewer head movements. It is unclear why the object in the cage did not cause a similar change in vigilance. Potential explanations could be a) the objects outside the cage were considerably larger than the star in the cage conveying different levels of threat [55]. b) The outside object may have required more assessment as it could only be explored from one direction, whereas the star could be approached from all directions providing more information about threats [56]. Last but not least, vigilance was lowest in the novel environment. This corroborates findings from an earlier study suggesting a tracking strategy to collect information about the new situation [22]. Few head movements mean that the bird is looking in one direction for longer. This allows collecting detailed information about this particular part of the environment [14]. While this is opposite to most studies, which find higher vigilance in unfamiliar situations [23, 52], it can be explained with the secrecy of this species as they sit quietly in a tree after arriving at a waterhole (own obs.).

Vigilance showed an age effect with a decrease in vigilance the older the bird, which was particularly visible in the oldest birds (over 4 years). Greater experience [4] in older birds may reduce head movements as they may be less aroused than younger individuals. Alternatively, the old birds may be less vigilant due to cognitive deterioration [21].

Males made fewer head movements than females. This can be interpreted as using a tracking strategy, whereby males track each other to avoid attack [14]. As they focus on an individual for longer, the frequency of head movements declines. Females, in contrast, may have generally scanned the environment moving the head frequently to cover all parts of the environment [14].

Finally, group composition affected vigilance. Individuals in mixed head colour morph groups were more vigilant than birds in pure red-headed or black-headed morph groups. Higher vigilance in mixed head colour morph groups was also found in an earlier study of this species [22]. Red-headed birds are more aggressive than black-headed Gouldian finches [40, 57, 58]. Black-headed birds may increase their vigilance to avoid aggression in the presence of red-headed birds [22]. An increase in social vigilance has been observed across taxa in more competitive situations [3, 5–7, 25]. Red-headed birds, in contrast, may watch black-headed birds to extract information about potential environmental threats as black-heads take greater risk [40] and investigate changes in the familiar environment faster [40, 45]. In both head colour morphs, greater social vigilance alongside environmental vigilance resulted in overall higher vigilance in mixed morph groups.

In conclusion, vigilance showed short-term contextual consistency but no long-term temporal consistency and some weak relationships to other personality traits supporting the risk-reward trade-off hypothesis. Vigilance was highly plastic across situations with the highest vigilance when changes occurred around important resources. Finally, vigilance was higher in mixed morph groups pointing to increased social vigilance in these settings.

## Supporting information

S1 Table Data about vigilance. (XLSX)
